# Unique microglia expression profile in developing white matter

**DOI:** 10.1186/s13104-019-4410-1

**Published:** 2019-07-01

**Authors:** Ori Staszewski, Nora Hagemeyer

**Affiliations:** Institute of Neuropathology, Medical Faculty, University of Freiburg, Breisacher Str. 64, 79106 Freiburg, Germany

**Keywords:** Amoeboid microglia, RNA sequencing, White matter, Grey matter

## Abstract

**Objective:**

Recently we demonstrated that amoeboid microglia in white matter regions are essential for proper oligodendrocyte homeostasis and myelinogenesis in the first postnatal week. Amoeboid microglia in the mouse corpus callosum change their activation profile within few days after postnatal day (P)7 with microglia of the cerebellum showing similar features. Here we expanded our previous transcriptional analysis and performed detailed bulk RNA sequencing of microglia from corpus callosum, cortex and cerebellum at P7, P10 and P42. The goal of this study was to identify a specific gene profile for both, white matter and grey matter microglia during development.

**Results:**

Microglia in white matter regions display unique characteristics in the first postnatal week of murine life. In both the corpus callosum and cerebellum microglia show amoeboid morphology and a similar transcription profile during development including high expression of genes related to priming of microglia, phagocytosis and migration at P7; characteristics which are already lost at P10. Together these data verify our previous transcriptional data obtained by microarray analysis and enable a more complete view into white matter and grey matter microglia at different developmental stages.

**Electronic supplementary material:**

The online version of this article (10.1186/s13104-019-4410-1) contains supplementary material, which is available to authorized users.

## Introduction

Microglia are the resident myeloid cells in the brain parenchyma with diverse functions during homeostasis and disease [1]. The accumulation of activated microglia with an amoeboid morphology along the ventricles and white matter regions within the first postnatal week was described as early as 1932 [2, 3]. This phenomenon was not restricted to rodents; in 1939 Kershman coined the term “fountain of microglia” for hot spots of activated microglia during human embryogenesis [4]. Many decades later this phenomena was confirmed by several studies [5–10]. Their function, however, has remained unknown. Recently we identified an essential role for amoeboid microglia in murine white matter regions for oligodendrocyte homeostasis and myelinogenesis during the first postnatal week [11], an observation which was replicated shortly after [12]. On the transcriptional level, microarray based analysis showed that microglia of the corpus callosum (CC) at postnatal day (P)7 are highly activated compared to cortical microglia. As we observed that the amoeboid phenotype rapidly changed within a few days after P7 and cerebellar microglia appeared to display very similar characteristics to callosal microglia, we proceeded here to analyze microglia during development in a more detailed way, by assessing P7, P10 and adult (P42) microglia from the CC, cortex (Cx) and cerebellum (Cb) with bulk RNA sequencing. Our data identify that the transcriptional profile of microglia in the CC and Cb at P7 and P10 is driven largely by age and much less by the specific brain region assayed and cortical microglia show a transcriptional profile different from white matter microglia. Finally, at P42 microglia from all brain regions appeared to share a common transcriptional pattern. These data suggest that early during postnatal development white matter microglia in all brain regions possess a unique transcriptional profile compared with grey matter that is lost during maturation.

## Main text

### Materials and methods

#### Mice

*Cx3cr1*^*GFP/WT*^ and wildtype (C57BL/6 J) mice were bred in-house under pathogen-free conditions. All animal experiments were approved by the Regierungspräsidium Freiburg, Germany, Section: Landwirtschaft, Ländlicher Raum, Veterinär- und Lebensmittelwesen. Application no. G17/55 and X-16/04.

#### Cell sorting and histology

Tissue samples were prepared as described before [11, 13]. Briefly, mice were lethally anesthetized and perfused. Brain tissue was fixed in 4% PFA for immunohistochemistry or brain regions were separated and homogenized for cell sorting.

Cell sorting was done on a MoFlo Astrios (Beckman Coulter, Krefeld, Germany). Live/Dead stain: Fixable Viability Dye eFluor^®^506 (eBioscience, San Diego, USA). Fc block: Fc-receptor blocking antibody CD16/CD32 (clone:2.4G2, BD Bioscience, Heidelberg, Germany). Antibodies used: anti-CD45 (clone:30-F11, eBioscience, San Diego, USA), anti-CD11b (clone:M1/70, eBioscience, San Diego, USA), anti-Gr1 (clone:RB6-8C5, Biolegend, Fell, Germany).

**Fig. 1:**
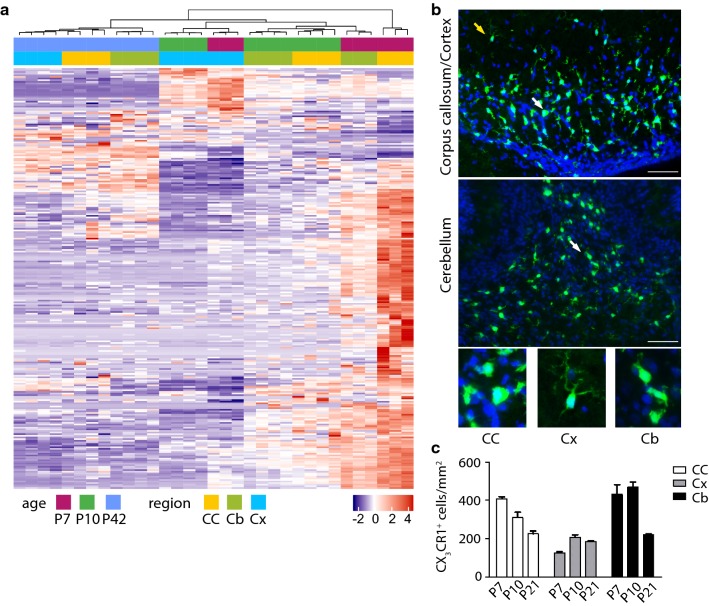
**a** Heatmap of the most regulated genes (all genes with absolute log2fold-change ≥ 1 and adjust p-value ≤ 0.01) in the corpus callosum (CC), cerebellum (Cb) and cortex (Cx) at different ages [postnatal day (P)7, 10, 42]. **b** Representative immunofluorescent images of *Cx3cr1*^*GFP/WT*^ mice at P7 showing microglia (green) with a ramified morphology in the cortex (yellow error) and amoeboid microglia (white errors) in the CC and Cb. Scale bar 200 µm; blue = DAPI. **c** Quantification of CX_3_CR1^+^ microglia in the CC, Cx and Cb at P7, P10 and P21; N = 3 mice per timepoint

For immunofluorescence staining tissues were treated as described before [11]. Briefly, mouse brains were fixed overnight, dehydrated and embedded in Tissue-Tek^®^O.C.T.TM Compound (Sakura Finetek Europ B.V., Netherlands). 12 µm cryosections were stained. Primary antibodies used: IBA-1 (1:500, Cat. No.: NB100-1028, Novus Biologicals, Wiesbaden, Germany), SPP1 (1:200, Cat. No.: ab8448, abcam, Berlin, Germany), CLEC7A (1:30, Cat. No.: mabg-mdect, InvivoGen, Toulouse, France) and CD206 (1:100, Cat. No.: MCA2235, Bio-Rad, Munich, Germany). Secondary antibodies used: Alexa Fluor 555, Alexa Fluor 568, Alexa Fluor 488, Alexa Fluor 647 1:500 (Life technologies, Darmstadt, Germany). Nuclei counterstaining: 4,6-diamidino-2-phenylindole (DAPI, 1:10,000, Cat. No.: 236276, Boehringer, Mannheim, Germany). Imaging was performed on the BZ-9000 Biorevo microscope (Keyence, Neu-Isenburg, Germany). N = 3–4/timepoint. Quantification of microglia (Fig. 1c) was performed on three parasagittal brain sections (every 10th section starting at the level of the rostral migratory stream) per animal (N = 3 mice).

#### RNA sequencing

FACS-sorted microglia were processed at the Genomics Core Facility “KFB-Center of Excellence for Fluorescent Bioanalytics” (University of Regensburg, Regensburg, Germany; http://www.kfb-regensburg.de). Library preparation and RNA sequencing was performed as described before [13]. Quality control of Fastq Files was done using FastQC [14]. Using the Star aligner (version 2.5.2b), reads were mapped to the GRCm38 mouse genome [15]. Read counts were acquired by the featureCounts (version 1.6.2) package with essentially standard settings. Specifically counts were combined at the gene level, multimapping and multi overlapping counts were discarded and reads were required to overlap by at least 1 base with an annotated feature. Differential gene expression analysis was done by the limma/voom (version 3.38) pipeline in R following the limma vignette [16–18]. Specifically genes with zero reads were discarded. Following counts were summarized for each gene over all samples and the lower third of genes were removed (averaging to about 1.5 counts per sample). After prefiltering the limma/voom pipeline was followed as described the vignette. Heatmaps were created using the ComplexHeatmaps R package [19]. Pathway Analysis was conducted using Ingenuity Pathway Analysis (IPA, Qiagen). For IPA analysis all genes with an adjust p-value of < 0.01 were retained and IPA analysis was performed with standard settings except that only experimentally observed interactions in mouse datasets were queried.

### Results

To investigate the expression profile of microglia during development in white and grey matter regions, we performed bulk RNA sequencing of fluorescent activated cell sorted (FACS) microglia (CD45^+^, CD11b^+^, Gr^−^) of the CC, Cx and Cb at postnatal day P7, P10 and P42. By analyzing the gene expression of microglia at these different ages and brain regions it became clear, that microglia of the CC and Cb display a similar expression profile. In these brain regions, the developmental stage had a higher impact on gene expression compared to brain region itself (Fig. 1a). In contrast, cortical microglia at P7 and P10 clustered separately from CC and Cb microglia at P7 and P10. This suggests that, in this instance, the brain region itself had higher impact on the gene profile of cortical microglia than did age. Finally, at P42, microglia of all brain regions investigated were quantitatively similar at the gene expression level. Next, using *Cx3cr1*^*GFP/WT*^ mice to assess microglia morphology using immunofluorescent analysis, we observed that microglia of the CC and Cb at P7 had an amoeboid shape, indicative of a high activation state of these cells (Fig. 1b white arrow). In contrast, cortical microglia had a ramified -not activated-morphology (Fig. 1b yellow arrow). Moreover, cell dynamics during development were very similar in the CC and Cb showing higher cell numbers at P7 and P10 compared to cortical microglia which decreased until P21 (Fig. 1c). Cortical microglia numbers increased up to P10 and showed a milder subsequent decline. Together these data indicate that developmental transcriptional profile, morphological phenotype, and cell dynamics are similar between microglia of the CC and Cb and different from cortical microglia.

**Fig. 2:**
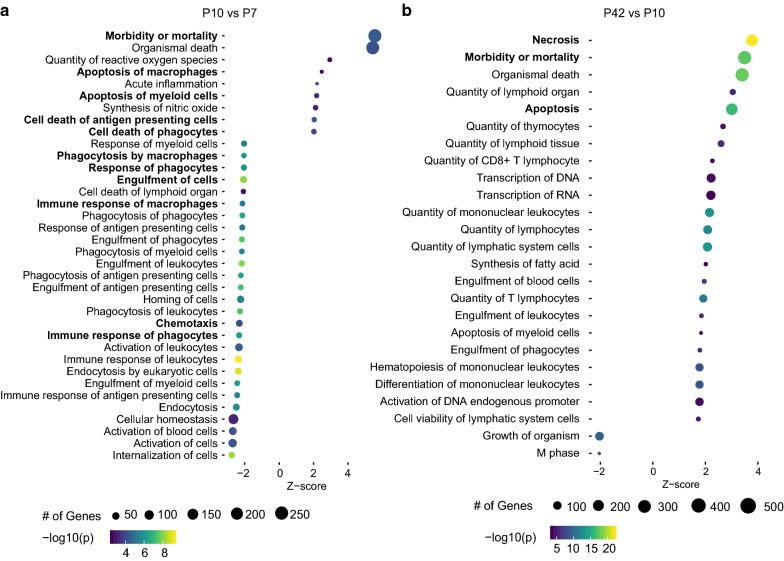
Pathway analysis of the most regulated genes in the corpus callosum at postnatal day (P)10 versus P7 (**a**) and P42 versus P10 (**b**)

**Fig. 3:**
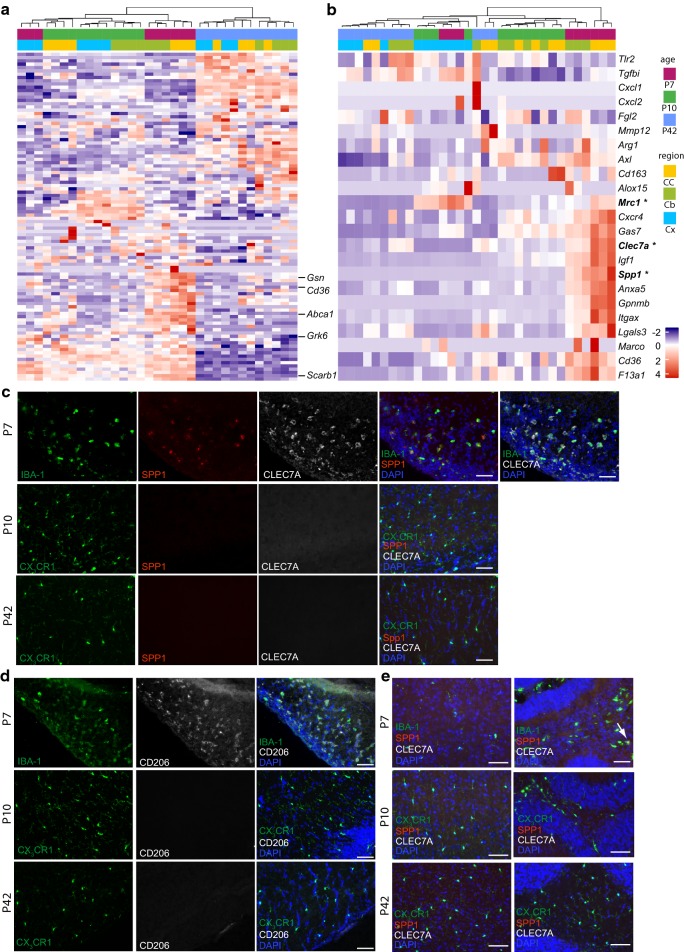
**a** Heatmap of regulated genes related to phagocytosis in the corpus callosum (CC), cerebellum (Cb) and cortex (Cx) at different ages [postnatal day (P)7, 10, 42]. **b** Heatmap of known priming genes in the CC, Cb and Cx at P7, P10 and P42. Asterisks highlighting genes confirmed on protein level; Representative immunofluorescence pictures of wildtype mice depicting SPP1 [red (**c**, **e**)], CLEC7a [white (**c**, **e**)] and CD206 [white (**d**)] expression in IBA-1^+^ microglia (green) in the CC (**c**, **d**), Cx (**e** left) and Cb (**e** right) at P7, P10 and P42. Scale bar 50 µm; blue = DAPI; arrow indicating IBA-1^+^/SPP1^+^ cell in Cb; N = 3–4 mice per timepoint

To more closely assess differences in gene expression over time, we performed pathway analyses, which demonstrated a higher expression of genes related to apoptosis at P10 compared to P7 in the CC, whereas at P7, genes related to phagocytosis and chemotaxis were highly upregulated compared to P10 (Fig. 2a). Interestingly, with further maturation (comparison of microglia at P42 to P10) mainly genes related to apoptosis and necrosis were highly expressed in the CC (Fig. 2b). A similar profile could be seen for microglia in the Cb and Cx related to apoptosis (data not shown), whereas genes related to phagocytosis (e.g. *Gsn*, *Cd36*, *Abca1*, *Grk6* and *Scarb1)* were not expressed as highly as in the CC and were not as strongly altered over time (Fig. 3a).

Finally, we carried out hierarchical clustering of known priming genes as *Axl*, *Mrc1*, *Gas7*, *Clec7a*, *Igf1*, *Spp1*, *Anxa5*, *Gpnmb*, *Itgax*, *Lgals3*, *Cd36* and *F13a1*, which suggested that these genes are highly upregulated in P7 CC microglia. In contrast, Cb microglia at P7 showed a somewhat lower upregulation of these genes, while no expression was observed in cortical microglia (Fig. 3b). At P10, upregulation of these genes was no longer observed in any of the three regions assessed. Finally, in agreement with the transcriptional assessment we observed clear expression of SPP1, CLEC7A and CD206 (*Mrc1*) in IBA-1^+^ microglia at P7 in the CC by immunohistochemistry (Fig. 3c, d) which was not detectable in the CC at P10 (Fig. 3c, d). Interestingly, in the Cb CLEC7A and CD206 were not expressed in white matter microglia at any timepoint, however single positive SPP1^+^ microglia or patches of SPP1^+^ microglia could only be found in the central area of the white matter at P7 (Fig. 3c right (arrow), Additional file 1: Figure S1b). No expression of SPP1, CLEC7A and CD206 could be found in microglia of the cortex at any timepoint (Fig. 3e left; Additional file 1: Figure S1a). These data, again, demonstrate similar phenotype of white matter microglia early in development and a strong transcriptional dynamic occurring over just a few days.

### Discussion

Here we have demonstrated a detailed RNA sequencing analysis of murine microglia of white and grey matter regions over different developmental stages. This complements our previous data which identified specific properties of a microglia subpopulation which is only present in the developing CC and cerebral white matter. First, we could demonstrate here that microglia of white matter regions, namely the CC and Cb, are closely related to each other at P7 and P10 compared to cortical grey matter microglia. Second, microglia in both CC and Cb undergo a change in their gene expression profile between P7 and P10, demonstrating rapid alterations specific to white matter microglia at these developmental stages. At P7, CC and Cb microglia display a distinct transcriptional profile, including genes related to phagocytosis and migration as well as priming of microglia described during aging and disease [20, 21]. Genes related to apoptosis and necrosis, instead, are highly expressed at P10, and further increased at P42. The unique phenotype of P7 CC microglia was further confirmed in assessment of SPP1, CLEC7A and CD206 protein immunoreactivity, which was uniquely present at this timepoint, while not present in the cortical microglia and only partly recapitulated (SPP1 expression) in microglia of the Cb. Third, in contrast to white matter microglia, gene transcription of cortical microglia was driven by the brain region itself and was therefore distinct from white matter microglia. Fourth, gene expression of microglia was similar across regions in the adult brain. These results indicate that white matter microglia, perhaps by migration and phagocytosis, may play a special role in local tissue dynamics around P7, impacting oligodendrocyte homeostasis and myelination. This role is likely restricted to a brief window of time, after which cell numbers decline through apoptosis. As maturation continues into adulthood, resident microglial populations develop a common homeostatic role across white and grey matter regions. It will be of interest to understand the responsible molecular mechanisms and functional consequences in future studies.

## Limitations

- Transcriptional data are presented without functional analyses.
- Juvenile stages could have been included.

## Additional file

**Additional file 1: Figure S1.** Representative immunofluorescent images of wildtype or *Cx3cr1*^*GFP/WT*^ mice presenting expression of SPP1 (red), CLEC7A (white) and CD206 (white) in IBA-1^+^ or CX_3_CR1^+^ microglia in the cortex (**a**) and cerebellum (**b**) at P7, P10 and P42. Scale bar 50 µm; blue = DAPI; arrow indicating IBA-1^+^/SPP1^+^ cell in Cb; N = 3–4 mice per timepoint.

## Data Availability

The main GEO accession number for RNAseq data: GSE132688

## Abbreviations

CC: corpus callosum
Cx: cortex
Cb: cerebellum
CNS: central nervous system
P: postnatal day
FACS: fluorescent activated cell sorting
