# Supplementary figures and images for: Unique microglia expression profile in developing white matter
Source: BMC Res Notes. 2019 Jul 1;12:367. doi: 10.1186/s13104-019-4410-1 (PMC6604453; doi:10.1186/s13104-019-4410-1)

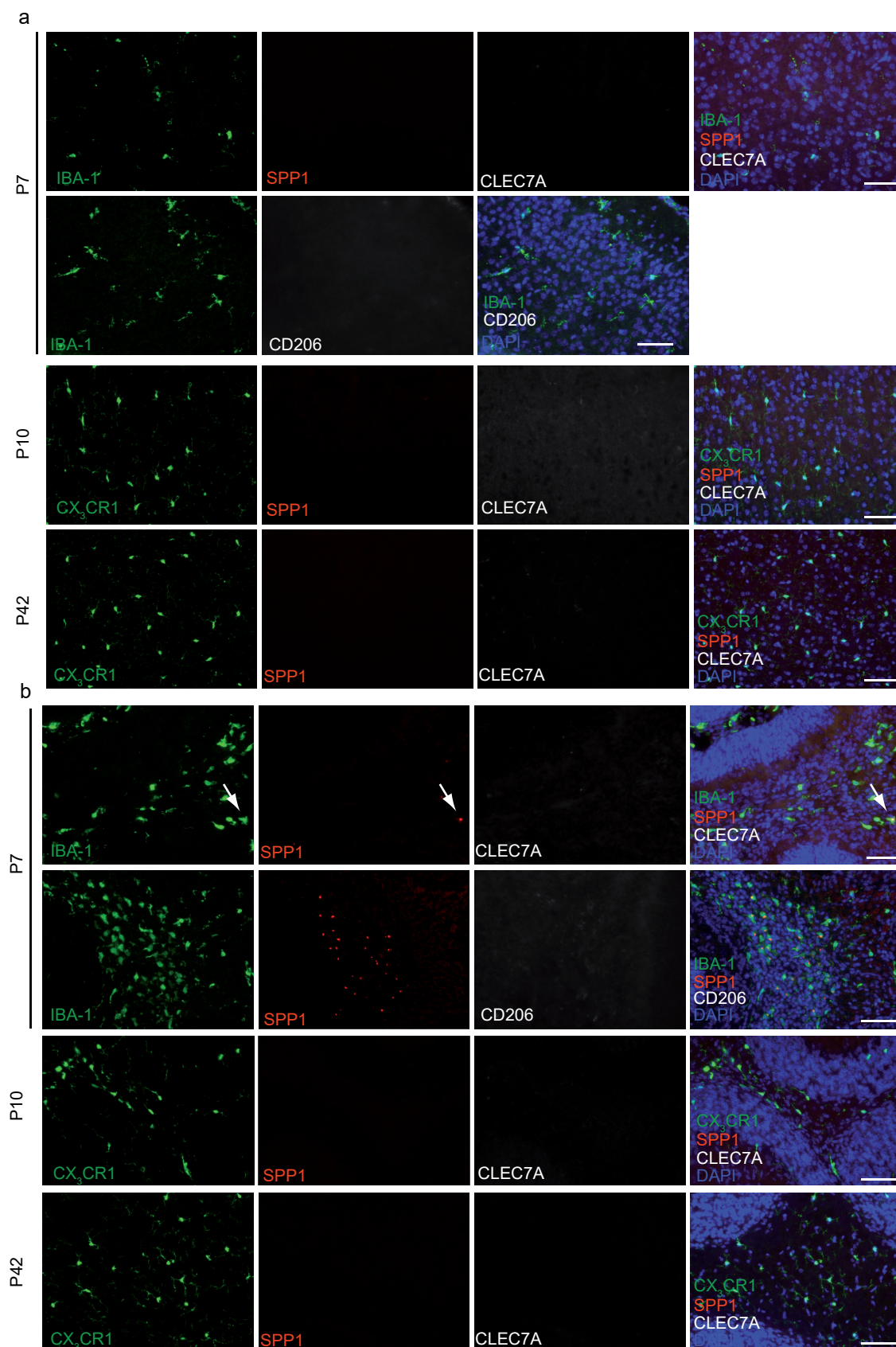

Additional Figure 1

Supplement: Supplementary file 1 — Additional file 1: Figure S1. Representative immunofluorescent images of wildtype or Cx3cr1GFP/WT mice presenting expression of SPP1 (red), CLEC7A (white) and CD206 (white) in IBA-1+ or CX3CR1+ microglia in the cortex (a) and cerebellum (b) at P7, P10 and P42. Scale bar 50 µm; blue = DAPI; arrow indicating IBA-1+/SPP1+ cell in Cb; N = 3–4 mice per timepoint. [file 13104_2019_4410_MOESM1_ESM.pdf]
